# Phenotypic adaptation of *Mycobacterium tuberculosis* to host-associated stressors that induce persister formation

**DOI:** 10.3389/fcimb.2022.956607

**Published:** 2022-09-27

**Authors:** Trisha Parbhoo, Jacoba M. Mouton, Samantha L. Sampson

**Affiliations:** Department of Science and Technology (DSI)-NRF Centre of Excellence for Biomedical Tuberculosis Research (CBTBR); South African Medical Research Council Centre (SAMRC) Centre for Tuberculosis Research; Division of Molecular Biology and Human Genetics, Faculty of Medicine and Health Sciences, Stellenbosch University, Cape Town, South Africa

**Keywords:** *Mycobacterium tuberculosis*, persistence, persisters, bacterial heterogeneity, host-pathogen interaction

## Abstract

*Mycobacterium tuberculosis* exhibits a remarkable ability to interfere with the host antimicrobial response. The pathogen exploits elaborate strategies to cope with diverse host-induced stressors by modulating its metabolism and physiological state to prolong survival and promote persistence in host tissues. Elucidating the adaptive strategies that *M. tuberculosis* employs during infection to enhance persistence is crucial to understanding how varying physiological states may differentially drive disease progression for effective management of these populations. To improve our understanding of the phenotypic adaptation of *M. tuberculosis*, we review the adaptive strategies employed by *M. tuberculosis* to sense and coordinate a physiological response following exposure to various host-associated stressors. We further highlight the use of animal models that can be exploited to replicate and investigate different aspects of the human response to infection, to elucidate the impact of the host environment and bacterial adaptive strategies contributing to the recalcitrance of infection.

## 1 Introduction

The interaction between a pathogen and its host is extremely complex. During infection with *Mycobacterium tuberculosis*, bacilli reside in various host microenvironments, which influence their physiological and metabolic state (Liu et al., 2016; Vijay et al., 2017a). As an adaptive mechanism in response to host-associated stressors, heterogeneous bacterial responses at a single-cell and population-wide level may drive the formation of phenotypically diverse subpopulations that enable survival in the host for prolonged periods. The majority of antibiotic-susceptible bacteria will succumb to host immune pressures, whilst a subpopulation of *M. tuberculosis* may transition into a persister state (Jain et al., 2016; Mouton et al., 2016). Persister bacteria are defined here as viable, but non- or slowly replicating, reversibly drug-tolerant subpopulations, with the potential to later resuscitate and cause active tuberculosis (TB) (Balaban et al., 2019; Moldoveanu et al., 2021). Persisters are able to survive exposure to high concentrations of antibiotics and/or host-associated stressors without known genetic mutations (Balaban et al., 2019). This is distinct from genetic resistance, which describes the ability of bacteria to grow at high concentrations of antibiotics through acquisition of resistance-conferring mutations. It is important to make the fine distinction between persister bacteria, and persistence of the infection. While the two are not mutually exclusive, in this review, the term “persistence” refers to failure to clear the infection.

*M. tuberculosis* has evolved to cope with diverse stressors in the host by manipulating specific host processes, in addition to modulating its metabolism to promote persister formation, enhance virulence, and ultimately prolong survival (persistence) within the host (Zimmermann et al., 2017; Huang et al., 2018; Russell et al., 2019). Improved knowledge regarding the strategies employed by *M. tuberculosis* to establish a persistent infection in the host, more specifically to sense and coordinate a physiological response, and trigger persister formation, is essential. The use of animal models (rabbits, guinea pigs, various mice and non-human primates) that recapitulate conditions experienced in the host, in combination with improved imaging technology is shedding light on the multifactorial mechanisms that contribute to *M. tuberculosis* persistence (Via et al., 2008; Lin et al., 2013; Gregg et al., 2018). Such animals display similarities in the pathophysiology and progression of infection compared to humans, however each model presents drawbacks, as highlighted in **Table 1**. Dependent on the animal model used, response to infection could differ, as observed in C3HeB/FeJ (Kramnik) versus BALB/c mice (Driver et al., 2012). The former model more accurately recapitulates certain aspects of human pathophysiology of infection progression where granuloma formation is observed. Further, C3HeB/FeJ mice display an increased susceptibility to *M. tuberculosis*, whilst the BALB/c model does not develop granuloma formation nor necrotic lesions (**Table 1**). However, the mycobacterial physiological state and adaptive mechanisms that promote a persister state during different stages of infection are largely unknown, and may be characterized by exploiting animal models that replicate different aspects of the human response to *M. tuberculosis* infection.

**Table 1:** *M. tuberculosis* progression of infection in animal models.

Associated clinical phenotype	Cynomolgus macaque	Rabbit	Guinea pig	Mice (BALB/C, C57BL/6)	Kramnik mice (C3HeB/FeJ)	Zebrafish Larvae model
Hypoxia	Present	Present	Present	Absent	Present	Present
Granuloma formation	Present	Present	Present	Absent	Present	Present
Caseous necrosis	Present	Present	Present	Absent	Present	Present
TAG accumulation	Present	Present	Present	Absent	Present	Present
Pulmonary pathology	Present	Present	Present	Present	Present	Absent
Dissemination (Intracellular and extracellular bacilli)	Present	Present	Present	Absent	Present	Present
Advantages	• Wide-spectrum of lesions observed, similar to humans • Latent infection observed	• Mid-range cost • Granuloma development and histopathology similar to humans	• Mid-range cost, easy to handle • Granuloma development and histopathology similar to humans	• Inexpensive, fast generation time (weeks) • Genetic variant strains available	• Inexpensive, fast generation time (weeks) • Genetic variant strains available • Lesion diversity observed, immune cells involved similar to humans • Latent infection observed • Evaluation of drug efficacy	• Easily bred and genetically manipulated, inexpensive • Small, fast grower • Transparency allows *in vivo* real-time visualization of infection
Disadvantages	• Expensive, difficult to handle, long generation time	• Limited availability of immunological tools • Does not establish latent infection	• Limited availability of immunological tools • Extremely vulnerable to infection, unable to tolerate certain drug classes • Does not establish latent infection	• Lack of granuloma structure and organization, no extracellular dissemination • Does not establish latent infection	• Increased frequency of drug resistance observed • Research unknown how immune susceptibility may be altered due to inactivation of the *Ipr1* gene	• Lung structure and lymphocytes are absent • *Mycobacterium marinum* used as a surrogate bacterium
References	Lin et al., 2006; Via et al., 2008	Via et al., 2008; Guerrini et al., 2018	Via et al., 2008	Via et al., 2008; Driver et al., 2012	Driver et al., 2012; Irwin et al., 2015	Commandeur et al., 2020; Liu et al., 2020

TAG, triacylglycerol; Ipr1, intracellular pathogen resistance-1.

Elucidating the impact of the host environment and bacterial adaptive strategies during infection may guide future research into the recalcitrance of infection to treatment. To improve our understanding of the physiological state of *M. tuberculosis* persisters, this review will focus on the phenotypic adaptation of *M. tuberculosis* in response to various host-associated stressors and the strategies exploited by *M. tuberculosis* to not only survive, but to enter a persister state and ultimately drive disease progression.

## 2 Host environment encountered by *M. tuberculosis*

Upon inhalation, *M. tuberculosis* bacilli are transported to lung alveoli where alveolar macrophages engulf bacilli into phagosomal compartments. Dependent on the polarization state of alveolar macrophages, bacterial replication may occur in these compartments (Refai et al., 2018; Huang et al., 2019). Alveolar macrophages respond rapidly to *M. tuberculosis* through the secretion of cytokines [Interleukin (IL)-12, IL-23, IL-18] to induce interferon-gamma (IFN-γ) and tumor necrosis factor alpha (TNF-α) production by natural killer and T-cells (de Martino et al., 2019). Following activation by IFN-γ and TNF-α, macrophages undergo a considerable phenotypic transformation, leading to the increased production of chemokines, cytokines and pro-angiogenic factors to assist with mycobacterial killing (Flynn and Chan, 2001). This exposes *M. tuberculosis* to a nutrient-limited macrophage environment, where the bacilli experience acid stress, hypoxia, and exposure to reactive oxygen and nitrogen intermediates (Manina et al., 2015). The unique metabolism and immune profile of macrophages is critical for promoting either a restrictive (M1 polarized) or permissive (M2 polarized) environment for *M. tuberculosis* (Marino et al., 2015; Huang et al., 2018; Pisu et al., 2020). These host environments have shown to alter *M. tuberculosis* growth and induce persister formation in *M. tuberculosis*, and will be discussed in the following sections.

A hallmark feature of *M. tuberculosis* infection is the formation of granulomas, organized immunological structures rich in immune cells that are recruited to the site of inflammation. Granulomas are composed of lymphocytes, neutrophils and macrophages; *M. tuberculosis* primarily inhabits the latter. Growth restriction and extracellular dissemination of *M. tuberculosis* is contained by granulomas, which acts in synergy with IFN-γ and TNF-α for structural maintenance of granulomas during early and late stages of infection (Mezouar et al., 2019). Mature macrophages may additionally undergo phenotypic alterations by fusing into multinucleated giant cells and differentiating into lipid-loaded foam cells (Sia and Rengarajan, 2019). Whilst *M. tuberculosis* is largely contained within the granuloma, the phagosome becomes highly vulnerable to rupture during later stages of infection, leading to cytosolic escape of *M. tuberculosis* (Simeone et al., 2015).

Changes in oxygen tension within the granuloma result in a shift to anaerobic metabolism whereby *M. tuberculosis* adapts to exploit alternative pathways and enzymes [such as regulation of the stringent response (Dutta et al., 2019), cytochrome bd oxidase, cytochrome bcc/aa3 (Mascolo and Bald, 2019), fumarate reductase, nitrate reductase, isocitrate lyase, and succinate] (**Supplementary Table 1**), which are functional at lower metabolic rates (Salina et al., 2014). These pathways are however regarded as energetically demanding, implying that alternative metabolites are being utilized for the maintenance of the proton motive force (PMF), adenosine triphosphate (ATP) and cofactor recycling (Prosser et al., 2017).

*M. tuberculosis* shifts its metabolism to the glyoxylate shunt when β-oxidation of fatty acids provides the main carbon and energy source (Hoff et al., 2011). Additionally, the presence of the non-proton-translocating electron donor, NADH dehydrogenase 2, along with nitrate reduction (NAD+ recycling) (Eoh and Rhee, 2013; Prosser et al., 2017) plays a key role in maintaining an energized membrane, ATP production and carbon precursors during hypoxia and nutrient limitation. These cofactors may additionally serve as a reserve of metabolic energy that can facilitate the extracellular spread of *M. tuberculosis* (Tan et al., 2010). The thick cell wall of *M. tuberculosis* additionally offers protection from reactive oxygen species (ROS) by scavenging oxygen radicals (Ehrt and Schnappinger, 2009). Remodeling of bacterial respiration additionally mitigates intracellular redox stress, as excessive accumulation of NADH/NADPH enhances ROS *via* the Fenton reaction, which can induce intracellular damage to bacterial proteins and DNA (Vilchèze et al., 2013; Vilchèze et al., 2017).

Further research to identify host factors manipulated by *M. tuberculosis* will enhance our understanding of how *M. tuberculosis* modulates the antimicrobial host response. The host-pathogen interaction may be crucial for determining the fate of infection, and whether specific pathways are manipulated for eradication of bacilli, containment and persistence, or bacterial dissemination.

### 2.1 Initial uptake of *M. tuberculosis* into phagosomes

**Figure 1:**
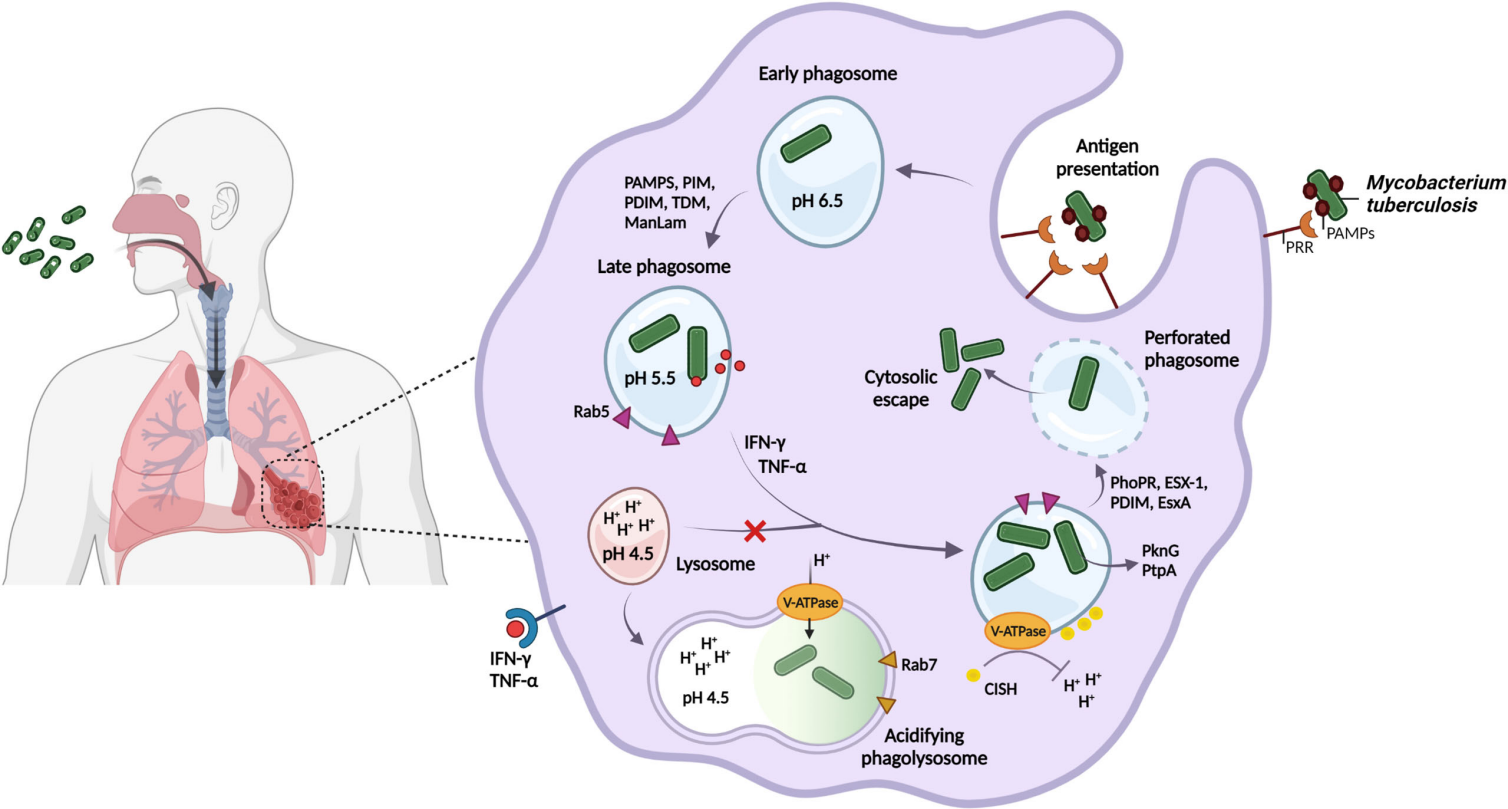
*M. tuberculosis* regulates phagosomal pH and blocks phagolysosome formation to avoid degradation by host acidic stressors and promote persister formation. *M. tuberculosis* is spread through the inhalation of aerosolized droplets that are released into the air when an infected individual coughs or sneezes. Host recognition of *M. tuberculosis*-specific PAMPs results in the internalization into the phagosome of alveolar macrophages for bacterial containment. *M. tuberculosis* may however interfere with host cellular processing and the host immune response by retaining its interaction with the host early phagosomal marker, Rab5, and by selectively engaging host receptors, as a strategy to enhance intracellular survival in the phagosome. Following host immune activation by IFN-ƴ and TNF-α, phagosomal acidification to pH 4.5 is proposed. *M. tuberculosis* inhibits phagolysosomal fusion and regulates the phagosomal pH by the V-ATPase proton pump to resist degradation by the acidic environment. *M. tuberculosis* recruits the host CISH protein to degrade V-ATPase for neutralization of the vacuole. Finally, ESX-1 and the mycobacterial cell envelope lipid PDIM, act in synergy to perforate the phagosome, which promotes cytosolic displacement of bacteria, and is a crucial step for intracellular persistence. Created with BioRender.com.

Phagocytosis of *M. tuberculosis* by macrophages plays a crucial role in pathogen detection, engulfment and immune regulation. Depending on the host cell receptors engaged (e.g. antigen- and pattern recognition receptors), specific microbial ligand sequences will be recognized, which could alter the initial interaction between host and pathogen, and subsequent intracellular processing (Kumar et al., 2019). Following internalization into the phagosome, *M. tuberculosis* exhibits a remarkable ability to interfere with the host antimicrobial response and inhibit phagosome-lysosome fusion (**Figure 1**, **Supplementary Table 1**).

*M. tuberculosis* possesses a diverse array of cell-surface ligands and attempts to manipulate host cell recognition and internalization through pathogen-associated molecular patterns (PAMPs, **Figure 1**), including trehalose-6,6-dimycolate (TDM, cord factor) (Patin et al., 2017; Lerner et al., 2020), phosphatidylinositol mannoside (PIM) and mannose-capped lipoarabinomannan (ManLam) (Torrelles and Schlesinger, 2010; Marakalala and Ndlovu, 2017; Queval et al., 2017a). Phagocytosis of beads coated with ManLam (Fratti et al., 2003; Kang et al., 2005; Shui et al., 2011) and TDM (Geisel et al., 2005; Axelrod et al., 2008) has been shown to interfere with phagosome maturation and promote granuloma formation, respectively.

TDM is a significant contributor to intracellular cording of *M. tuberculosis*, whereby large lipid pellicles aggregate on the cell surface, preventing cytosolic recognition and phagocytosis into space-constrained vacuoles (Lerner et al., 2020). In contrast, dead bacilli are rapidly transported to lysosomes for degradation (Malik et al., 2000; Kyei et al., 2006). Since host immune cells express varying combinations of receptors, their interaction with PAMPS may differ (Liu et al., 2017; Dubé et al., 2021). *M. tuberculosis* may preferentially target specific host cell receptors to manipulate the host immune response, however, the mechanism of how manipulation by PAMPs influences macrophage metabolism, the delay of phagosome-lysosome fusion and subsequent intracellular processing, remains to be explored.

### 2.2 Phagosome acidification

Phagosome acidification represents a dynamic antimicrobial defense strategy used by the host, and despite *M. tuberculosis* being sensitive to high acidity (low pH), the pathogen appears to resist being killed in macrophages by maintaining its intra-bacterial pH (Vandal et al., 2008; Levitte et al., 2016). Several mechanisms that assist in neutralizing phagosomal pH have been proposed (**Figure 1**), including membrane proteins, proton pumps, ammonia production, amino acid decarboxylation and cell wall modifications (Vandal et al., 2008; Singh et al., 2019).

During phagosomal maturation, highly conserved vacuolar-type ATPase (V-ATPase) proton pumps are rapidly recruited to the phagosomal membrane to promote acidification. *M. tuberculosis* has however developed the ability to target and trigger degradation of the V-ATPase complex by inducing the expression of the host cytokine-inducible SH2-containing protein (CISH) (Queval et al., 2017b). This allows the phagosomal pH to remain above that required for the activity of lysosomal digestive enzymes and ROS (Pauwels et al., 2017; Baker et al., 2019).

**Figure 2:**
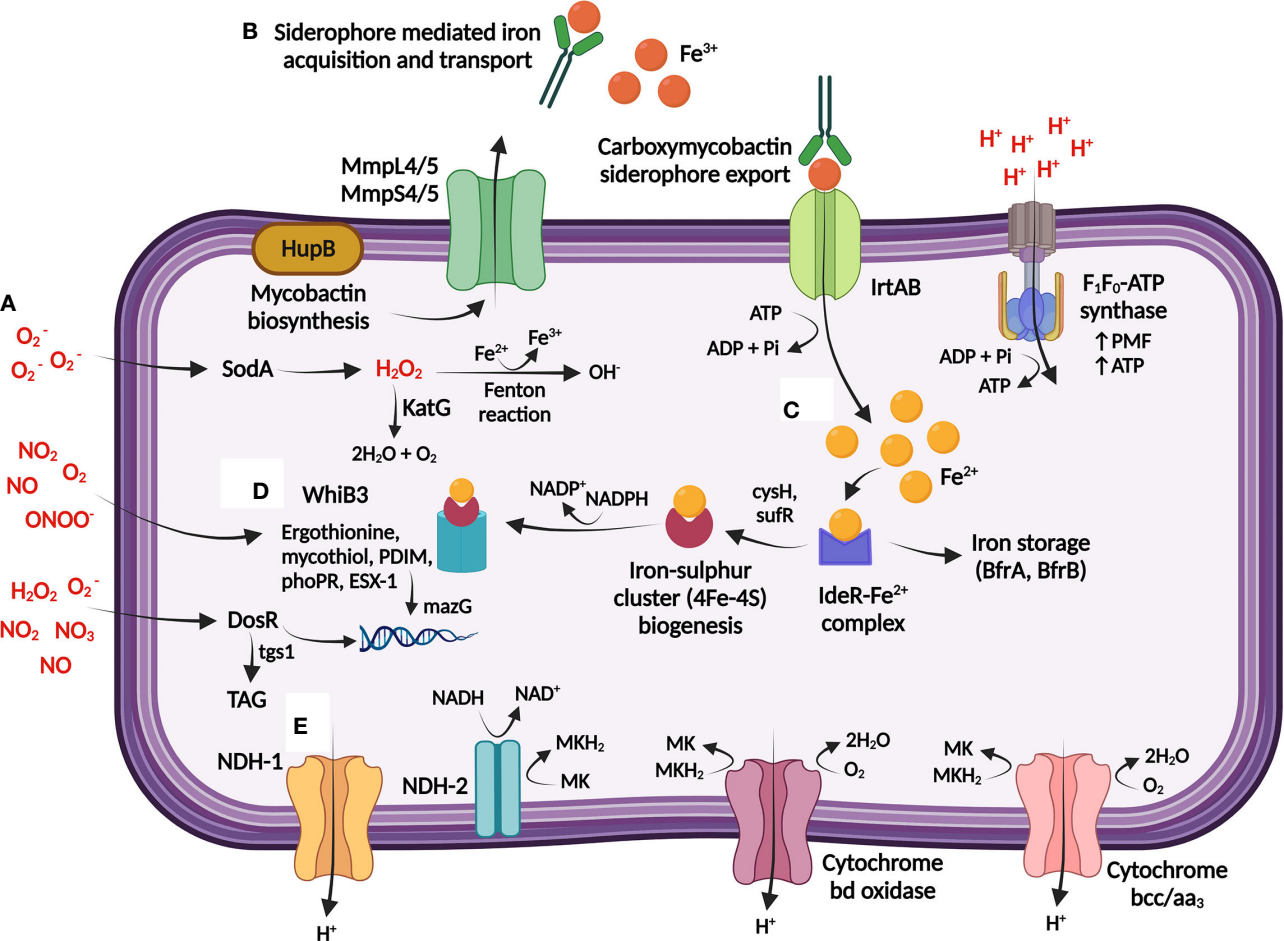
*M. tuberculosis* maintains bioenergetic metabolism to ensure redox homeostasis during persistence. **(A)** The host generates toxic reactive oxygen species (ROS) and reactive nitrogen species (RNS) [shown in red] through single-electron reactions, which can result in damage to *M. tuberculosis* DNA and proteins. **(B)** Redox homeostasis is maintained through the sensing and regulation of iron, obtained from the host *via* Fe^3+^-specific iron scavenging siderophores that is imported into *M. tuberculosis* by the ABC exporter, IrtAB. **(C)** Iron is either stored or utilized directly for generation of iron sulphur clusters (4Fe-4S), and further encoded to 4Fe-4S redox sensor proteins by WhiB3. **(D)** WhiB3 produces detoxification buffers (ergothionine and mycothiol) and in combination with DosR directs the reducing equivalents produced during β-oxidation of fatty acids to produce cell wall lipids and protect DNA from oxidation. DosR further regulates lipid storage within *M. tuberculosis* through the regulation of TAG, providing a lipid rich nutrient source. **(E)** Proton balance during redox homeostasis is maintained through regulation of internal protons which can be utilised for the production of menaquinol (MKH_2_) [via NDH-1/NDH-2], which is subsequently transferred to the respiratory complexes (cytochrome bd and cytochrome bcc/aa_3_) to maintain the PMF and ATP production *via* F_1_F_0_-ATP synthase. TAG: triacylglycerol; MK: Menaquinone; NDH: NADH dehydrogenase; Fe^3+^: ferric ion; Fe^2+^: ferrous ion; ${\text{O}}_{2}^{-}$ : superoxide; NO: nitric oxide; NO_2_: nitrogen dioxide; ONOO^-^: peroxynitrite; H_2_O_2_: hydrogen peroxide; MmpL: mycobacterial membrane protein large; MmpS: mycobacterial membrane protein small. Created with BioRender.com.

The ability of *M. tuberculosis* to rapidly sense and respond to the external pH is crucial for pathogenesis, and involves strong induction of the PhoPR two-component regulatory system *in vitro* and in macrophages (Abramovitch et al., 2011). PhoPR is further required for intracellular pathogenesis and redox homeostasis, such as induction of the type VII secretion system ESX-1, regulation of heat shock-responsive genes (Sevalkar et al., 2019), and lipid biosynthesis regulated by the cytoplasmic redox sensor, WhiB3 (Johnson et al., 2015; Mehta et al., 2016; Feng et al., 2018) (**Figures 1**, **2**).

Transport of the ESX-1-dependent 6-kDa early secretory antigenic target (ESAT-6/EsxA) to the host cytosol (Simeone et al., 2015; Conrad et al., 2017) acts synergistically with the mycobacterial cell envelope lipid phthiocerol dimycocerosate (PDIM) in perforating the phagosome (Augenstreich et al., 2017; Barczak et al., 2017; Quigley et al., 2017; Lerner et al., 2020) and preventing recruitment of V-ATPase, thereby neutralizing the vacuole (Astarie-Dequeker et al., 2009; Passemar et al., 2014). Further membrane damage by continued perforation or complete rupture of the phagosome will allow translocation of the bacilli to the host cytosol (Jamwal et al., 2016; Schnettger et al., 2017) (**Figure 1**). This is crucial for intracellular persistence, as PDIM or *esxA* mutants failed to perforate the phagosomal membrane, possessed altered PAMPs, and were attenuated in human monocyte-derived macrophages (hMDMs) (Augenstreich et al., 2017). This suggests that remodeling of the cell envelope and central metabolism pathways may improve intracellular survival of persisters during acid stress by controlling the flux of lipid precursors and reducing equivalents (Baker and Abramovitch, 2018; Singh et al., 2019).

### 2.3 Oxidative and nitrosative stress

Host immune activation of macrophages stimulates multiple antimicrobial defense pathways, exposing *M. tuberculosis* to an environment where hydrolases, reactive nitrogen species (RNS), and ROS effectively function (Ehrt et al., 2001; Jamaati et al., 2017). *M. tuberculosis* effectively resists degradation by these reactive molecules by activating detoxification and redox buffering mechanisms to maintain bioenergetic homeostasis (Saini et al., 2016) (**Figure 2**; **Supplementary Table 1**).

Exposure to toxic nitric oxides slows bacterial growth by competing for oxygen binding, and in the process, reversibly inhibits cytochrome c oxidase, and thus aerobic respiration (Voskuil et al., 2003). *M. tuberculosis* metabolically adapts to acquire nitrate *via* oxidation of nitric oxide, thereby restoring survival due to maintenance of the PMF and ATP (Sohaskey, 2008; Tan et al., 2010). Induction of cytochrome *bd* by *M. tuberculosis* additionally plays a respiratory protective role against ROS/RNS by scavenging oxygen radicals upon breakdown of oxidative species (Small et al., 2013; Boot et al., 2017).

Host maintenance of the oxidative balance in the lung is controlled *via* antioxidant mechanisms, and *M. tuberculosis* utilizes the ROS scavenging enzymes, superoxide dismutase (SOD) and catalase-peroxidase (KatG) to degrade superoxide ${\text{(O}}_{2}^{-})$ to water and molecular oxygen to neutralize the free radicals generated in the host (Kumar et al., 2011) (**Figure 2**). Subsequent downregulation of ATP synthase genes may further slow bacterial growth by restricting proton translocation into the cytoplasm, thereby replenishing pools of oxidized cofactors to maintain redox and pH homeostasis (Baker et al., 2014; Baker et al., 2019). These metabolic adaptations prevent an excess of RNS/ROS, which may trigger damage to proteins, lipids and nucleic acids.

The pyrimidine-specific housekeeping enzyme, MazG, prevents DNA mutagenesis by specifically degrading and preventing the incorporation of oxidized deoxynucleotides into genomic DNA (Lyu et al., 2013; Shi et al., 2019) (**Figure 2**). Deletion of *mazG* decreases the NADH/NAD+ redox balance towards an oxidizing state, resulting in DNA instability, disruption to pyrimidine metabolism, and hindrance to iron and carbon uptake *in vitro* (Shi et al., 2019) and in mice (Lyu et al., 2013).

Free intracellular iron within *M. tuberculosis* catalyses ROS formation, thus homeostasis of iron is maintained by *M. tuberculosis* in a tightly regulated manner *via* the iron-dependent regulator (IdeR) (Rodriguez et al., 2002; Pandey and Rodriguez, 2014). Mycobactin and cysteine biosynthesis is maintained by *M. tuberculosis* to replace iron (Rodriguez et al., 2002) and sulphur (Song and Niederweis, 2012), respectively, for reconstruction of the damaged iron-sulphur clusters (**Figure 2**). This is essential for persister adaptation as iron-sulphur clusters are highly susceptible to oxidative and nitrosative stress as they undergo various oxidation-reduction reactions (Pandey et al., 2018). Paradoxically, iron uptake and cysteine synthesis promote DNA damage by forming reactive and damaging hydroxyl radicals *via* the Fenton reaction (**Figure 2**). However, the requirement of these proteins to repair damage appears to outweigh the negative effects of intermediate hydrogen peroxide and nitric oxide levels (Ford et al., 2011; Voskuil et al., 2011; Lyu et al., 2013).

Protection against ROS (Bhaskar et al., 2014) and nutrient starvation (Richard-Greenblatt et al., 2015; Saini et al., 2016) respectively involves WhiB3-dependent upregulation of the *M. tuberculosis* redox buffers, mycothiol and ergothioneine (**Figure 2**; **Supplementary Table 1**). These low molecular weight buffers maintain redox and bioenergetic homeostasis, including consumption of excess NADH/NADPH (Singh et al., 2009; Saini et al., 2016; Mehta and Singh, 2019). Lipid anabolism may therefore counteract reductive stress by oxidizing NADH/NADPH, as *phoP* mutants displayed a structurally altered cell envelope consisting of limited methyl branched fatty acids and diminished acid-fast staining (Walters et al., 2006).

### 2.4 Hypoxic environment within granulomas

**Figure 3:**
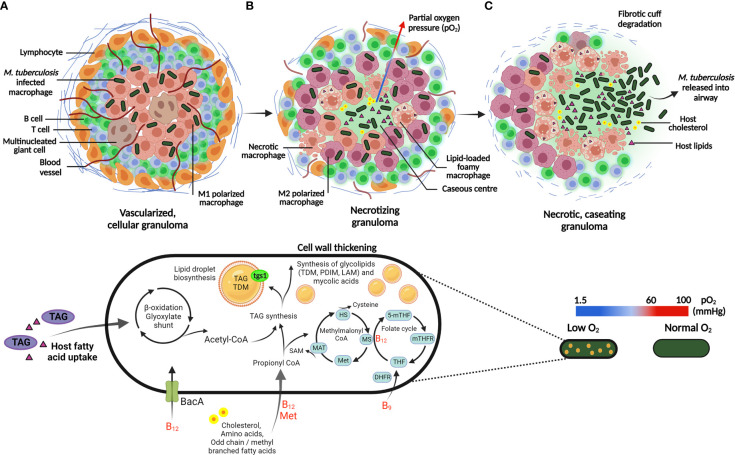
Adaptation of *M. tuberculosis* to the host granuloma environment and associated stressors. **(A)** *M. tuberculosis* is phagocytosed by alveolar macrophages and contained within the center of the granuloma, which is an organized aggregate of immune cells recruited to the site of inflammation. The pro-inflammatory cytokines, IFN-y and TNF-α activate macrophages (M1 polarized; pink macrophages), which is essential for the control of infection. Following host immune activation, recruitment of lymphocytes, largely comprising of T and B cells, surround the periphery of the granuloma. **(B)** Mature granulomas develop heterogeneous vasculature, displaying a gradient of pro-inflammatory host cells (M1 and M2 polarized; pink and purple macrophages, respectively) and a decreasing oxygen gradient toward the caseum. In response to hypoxia, *M. tuberculosis* exploit lipid-loaded foamy macrophages to store host TAG during persistence. Host fatty acids and cholesterol are metabolized by the β-oxidation and methylmalonyl CoA pathway respectively, which generates a nutritional carbon source that can be stored in lipid droplets or utilized to generate mycobacterial cell wall components. Essential vitamins utilized in the respective metabolic pathways (shown in red) are acquired by *M. tuberculosis* from the host environment *via* the ABC-transporter, BacA. **(C)** Necrotic macrophages contributes to an accumulation of lipid debris, resulting in central caseation in mature granulomas. Eventual rupture of the granuloma leads to extracellular dissemination of *M. tuberculosis.* HS: homocysteine; MS: methionine synthase; Met: methionine; MAT: methionine adenosyltransferase; 5-mTHF: 5-methyltetrahydrofolate; mTHFR: methylenetetrahydrofolate reductase; THF: tetrahydrofolate; DHFR: dihydrofolate reductase. Created with BioRender.com.

As granulomas mature, gradual accumulation of necrotic cellular debris from lysed or damaged host and bacterial cells forms a caseous core (Hoff et al., 2011). Enlargement of the caseum during advanced stages of active TB disease compresses the adjacent lung tissue, damaging the vasculature in the process, and distributing extracellular *M. tuberculosis* to the caseous center (Blanc et al., 2018; Dartois, 2014). The reduced vascularization additionally decreases oxygen availability (Datta et al., 2016), and in response, *M. tuberculosis* undergoes rapid and substantial metabolic and phenotypic adaptations (Qualls and Murray, 2016; Sershen et al., 2016) (**Figure 3**; **Supplementary Table 1**).

Foamy macrophages in necrotic regions accumulate triacylglycerol (TAG) within lipid droplets, a process exploited by *M. tuberculosis* to store host TAG intracellularly, providing a lipid-rich microenvironment for the persistence of *M. tuberculosis* during hypoxia (Guerrini et al., 2018; Del Portillo et al., 2019). Additionally, *M. tuberculosis* expression of triacylglycerol synthase (*tgs1*) induces lipid body formation in persisters, whereby lipid body-positive *M. tuberculosis* has been detected in sputum prior to the onset of treatment (**Figure 3**). The respiratory state of these bacilli displayed a shift from oxygen electron transfer to anaerobic respiration, thus challenging the belief that all bacilli in sputum respire aerobically (Garton et al., 2008).

ATP production in aerobic and anaerobic conditions is responsible for generating the membrane potential and proton concentration gradient that drives the PMF (Rao et al., 2008; Reichlen et al., 2017). Inhibitors of the membrane potential and proton concentration gradient are cidal towards persisters and lead to a loss in viability, suggesting that the cytoplasmic membrane of non-replicating hypoxic *M. tuberculosis*, induced in the Wayne model, is energized (Rao et al., 2008). The mechanisms involved in the maintenance of ATP suggest this process as essential for the survival of non-replicating *M. tuberculosis* under hypoxic conditions.

### 2.5 Adaptation during nutrient deprivation

Emerging evidence suggests that the active depletion of nutrients by the host immune system may create a nutrient-scarce environment within the phagosome (Appelberg, 2006; Eisenreich et al., 2013). Restricted access to transition metals (Kehl-Fie and Skaar, 2010; Hood and Skaar, 2012), carbon (Pandey and Sassetti, 2008; MacMicking, 2014), and amino acids (Zhang et al., 2013; MacMicking, 2014) drives the downregulation of key metabolism and replication machinery, possibly leading to auxotrophy in *M. tuberculosis*.

Auxotrophy is described as the inability of an organism to synthesize a specific metabolite required for its growth. *M. tuberculosis* auxotrophs for methionine (Berney et al., 2015), threonine (Hasenoehrl et al., 2019), lysine (Pavelka et al., 2003), leucine (Hondalus et al., 2000), and arginine (Tiwari et al., 2018) are severely attenuated *in vivo* due to their inability to scavenge these metabolites from the host. The *M. tuberculosis* stringent response pathway RelMtb, initiates production of the alarmones (p)ppGpp during amino acid deprivation, hypoxia and oxidative stress (Dutta et al., 2019). (p)ppGpp assists in enhancing survival during nutrient starvation in mice (Dahl et al., 2003) and guinea pigs (Klinkenberg et al., 2010; Thayil et al., 2011; Dutta et al., 2019) by inhibiting RNA synthesis for conservation of energy. Therefore, *M. tuberculosis* is incapable of entering persistence upon disruption to its stringent response (Dutta et al., 2019).

Certain metabolic processes are energetically expensive; *M. tuberculosis* has however developed elaborate strategies to synthesize or sequester essential host nutrients to satisfy its bioenergetic and biosynthetic requirements (Eisenreich et al., 2013). *M. tuberculosis* additionally exploits specific nutrients or metabolic processes that facilitate adaptation to persistence (**Supplementary Table 1**, **Supplementary Table 2**).

#### 2.5.1 Essential amino acids act as structural components, co-factors and building blocks

Amino acids represent a critical source of nutrients used to fuel central metabolic pathways, and many pathogens are required to scavenge them in the host. The host compartments either restrict metabolite availability, or *M. tuberculosis* attempts to evade host detection by remaining metabolically independent from the host (Berney et al., 2015). *M. tuberculosis* has evolved to synthesize all 20 proteinogenic amino acids (Cole et al., 1998), of which certain amino acids are rapidly metabolized as carbon, sulphur, nitrogen or energy sources, whilst others are stored. Amino acids may additionally function as both carbon and nitrogen sources, indicating that the pathways required for their degradation and assimilation could differ (Lofthouse et al., 2013). *M. tuberculosis* furthermore exploits amino acids for their ability to provide cofactors for generating intermediate molecules or proteins, which are integral components for metabolic adaptation (**Supplementary Table 2**).

Amino acids acquired from the macrophage, such as alanine, glutamate and asparagine/aspartate contribute to the intracellular nutrition of *M. tuberculosis* (Beste et al., 2013). Alanine is an essential structural component of peptidoglycan (Beste et al., 2013); cell wall homeostasis is maintained by *M. tuberculosis* during persistence by conversion of L-alanine to D-alanine, whilst an impaired conversion severely restricts intracellular growth in bone marrow-derived macrophages (BMDMs) and mice (Awasthy et al., 2012). During persistence, regulation of the aspartate pathway is involved in essential cell wall processes, such as biosynthesis of the cell wall cofactor, S-adenosylmethionine (SAM), generation of the essential amino acids methionine, isoleucine, threonine and lysine, and the precursor metabolite homoserine (Hasenoehrl et al., 2019). The majority of the methionine pool is converted to SAM *via* the aspartate pathway (Berney et al., 2015; Hasenoehrl et al., 2019), therefore it is suggested that metabolic regulation of the aspartate pathway is essential for *M. tuberculosis* persistence.

#### 2.5.2 Carbon and lipid metabolism: exploitation of the host niche

*M. tuberculosis* preferentially utilizes carbon-based metabolism during persistence. C-flux metabolism analysis indicates the adaptation of intracellular *M. tuberculosis* to simultaneously co-catabolize multiple carbon sources, including amino acids, carbon dioxide, vitamins and fatty acids; the latter derived from host lipids and cholesterol (de Carvalho et al., 2010; Beste et al., 2013; Noy et al., 2016; Borah et al., 2021). *M. tuberculosis* possesses the unusual ability to metabolize host fatty acids as substrates for β-oxidation. This yields substrates required for production of mycolic acids, or enables assimilation directly into TAG and phospholipids for maintenance of the cytoplasmic membrane integrity (Pandey and Sassetti, 2008) (**Figure 2**).

Lipid storage becomes the primary energy-conserving metabolic process in *M. tuberculosis* persisters, whereby intracellular accumulation of TAG has been observed (Russell et al., 2009; Daniel et al., 2011; Maurya et al., 2018). *M. tuberculosis* persisters may rapidly accumulate host lipids to form lipid bodies in resting macrophages, whilst access to lipid bodies is limited once macrophages are IFN-ƴ activated due to production of host protective eicosanoids (Knight et al., 2018). Instead, *M. tuberculosis* accumulates lipids from host macrophage lipid bodies stimulated by extracellular free fatty acids or by hypoxia (Daniel et al., 2011; Knight et al., 2018). Observed within necrotic lesions, lipid-loaded foamy macrophages further represent a lipid-rich niche whereby host cholesterol serves as a carbon source for persisters (Pandey and Sassetti, 2008; Russell et al., 2009; Singh et al., 2012) (**Figure 3**).

#### 2.5.3 Vitamins and cofactors central to nucleic acid and amino acid metabolism

β-oxidation of fatty acids accumulated by *M. tuberculosis*, generates a pool of coenzyme-A carriers and carbon units; even-chain fatty acids are degraded to acetyl coenzyme A (acetyl-CoA) *via* oxidation by the glyoxylate shunt to replenish central metabolites, whilst odd-chain fatty acids are degraded to propionyl-CoA (Warner et al., 2007; Savvi et al., 2008). Detoxification of the latter *via* the methylmalonyl-CoA and methyl citrate cycle is a vitamin B_12_-dependent and B_12_-independent mechanism, respectively (**Figure 3**). Additionally, MetH and MetE involved in methionine biosynthesis encode vitamin B_12_-dependent and B_12_-independent methionine synthase, respectively (Warner et al., 2007; Savvi et al., 2008). This indicates the essentiality of methionine biosynthesis, as *M. tuberculosis* may need to rapidly detect vitamin B_12_ bioavailability for prevention of toxic accumulation of propionyl-CoA (**Figure 3**). It is proposed that *M. tuberculosis* scavenges vitamin B_12_ from the host *via* the ATP-binding cassette (ABC) transporter, BacA (Gopinath et al., 2013). Deletion of *bacA* is speculated to contribute to the accumulation of cholesterol-rich lipid bodies (Gopinath et al., 2013), and since cholesterol provides a source of propionyl-CoA, efficient assimilation of propionyl-CoA is required for effective cholesterol metabolism (**Supplementary Table 2**).

An imbalance in vitamin B_12_ and methionine synthase is often associated with disruption to folate (Vitamin B_9_) metabolism, as methionine synthase utilizes 5-methyltetrahydrofolate (5-MTHF) as a one-carbon (methyl group) donor to catalyze the methylation of homocysteine to methionine (Nixon et al., 2014). This is essential for production of purines, thymidine, methionine, glycine, serine, homocysteine and SAM. Inhibitors of folate synthesis exhibit distinct metabolic disruptions to methionine and SAM *in vitro*, with an upregulation of genes associated with DNA repair (Nixon et al., 2014). Disruption to SAM may be attributed to imbalances in DNA methylation, thereby introducing single nucleotide polymorphisms (SNPs) and DNA damage. This subsequently starved cells of crucial reduced folate precursors critical for the synthesis of DNA, RNA and protein (Chakraborty et al., 2013; Zheng et al., 2013; Minato et al., 2015). It has been suggested that methionine-mediated antagonism of anti-folate drugs may enhance methylation by increasing SAM abundance (Howe et al., 2018). This could be an adaptive process; however, the antagonistic mechanism has yet to be characterized. This further confirms that metabolic remodeling may offer an advantageous adaptation mechanism for conserving carbon and energy sources for prolonged persistence and possibly resuscitation.

#### 2.5.4 Scavenging of host iron is tightly regulated

Persistence of *M. tuberculosis* during iron starvation is controlled by strict regulation of iron utilization and storage *via* the iron-dependent regulator IdeR; following acquisition, iron not immediately utilized is stored in the host as protein-bound iron in the form of ferritin to prevent the generation of toxic free radicals *via* the Fenton reaction (Pandey and Rodriguez, 2014) (**Figure 2**). Key cellular processes such as electron transfer, DNA replication and repair, and regulation of gene expression in response to ROS/RNS are dependent on iron and iron-sulphur clusters as a cofactor (Rodriguez and Smith, 2003). Iron is however not only restricted within granulomas, but the abundance of free iron is scarce due to its low solubility (Kurthkoti et al., 2017).

*M. tuberculosis* strongly promotes sequestration of iron following macrophage uptake (Abreu et al., 2018), and in necrotic and cavitary granulomas, where *M. tuberculosis* synthesizes and secretes high-affinity Fe^3+^-specific siderophores (mycobactins) to actively compete for host iron (Kurthkoti et al., 2017) (**Figure 2**, **Supplementary Table 2**). This process is further dependent on the ESX-3 secretion system, as *M. tuberculosis* lacking the ESX-3 secretion system are defective in acquisition of bound iron from siderophores, and display severely impaired growth in THP-1 macrophages (Serafini et al., 2013), murine macrophages (Siegrist et al., 2009), and human granulomas (Kurthkoti et al., 2017).

Upon iron starvation, the *M. tuberculosis* heme enzyme KatG is downregulated, therefore reduced bioactivation of isoniazid (INH) likely mediates enhanced tolerance, contributing to antibiotic-tolerant persister formation (Kurthkoti et al., 2017). Metabolic reprogramming of iron-starved bacilli displayed repression in the majority of enzyme-encoding genes from the citric acid cycle (TCA) and oxidative phosphorylation, since these pathways require iron-sulphur clusters or heme proteins (Kurthkoti et al., 2017). DNA repair genes, including iron-sulphur cluster repair proteins were upregulated, poising bacteria to respond to oxidative damage (Tyagi et al., 2015). Additionally, biosynthesis of most amino acids decreased, with exception to lysine (Kurthkoti et al., 2017), which is the backbone of siderophore biosynthesis (McMahon et al., 2012).

#### 2.5.5 Sulphur metabolism is intricately tied to oxidative stress

Sulphur assimilation pathways in *M. tuberculosis* produce reduced sulphur-containing metabolites, such as cysteine and methionine, and have been shown to play a critical role in facilitating bacterial adaptation and protection during persistence (Rhee et al., 2005; Senaratne et al., 2006), and upon exposure to oxidative stress and hypoxia (Pinto et al., 2004; Voskuil et al., 2011). Controlled by *cys* genes, sulphur is required to maintain redox reactions through iron-sulphur clusters, translation initiation, methylation of DNA and RNA, biotin and menaquinone synthesis, and mycolic acid modification by SAM-dependent methyltransferases (Hasenoehrl et al., 2019).

Disruption in CysH renders *M. tuberculosis* unable to utilize inorganic sulphate for the synthesis of the cytosolic reducing buffer, mycothiol (Senaratne et al., 2006), which serves in detoxification of bactericidal agents and host oxidative damage (Buchmeier et al., 2003; Senaratne et al., 2006; Mishra et al., 2019). Perturbation in mycothiol redox homeostasis elevated endogenous ROS, inducing a long-lasting and irreversible oxidative shift in *M. tuberculosis* (Tyagi et al., 2015). Adaptation may have favored the release of secretory antioxidants in the phagosome to neutralize exogenous oxidative stress as a defense mechanism, playing a role in facilitating persistence (Tyagi et al., 2015).

To further neutralize host-mediated oxidative stress, *M. tuberculosis* overexpresses cysteine desulfurase to repair oxidatively damaged iron-sulphur clusters (Ayala-Castro et al., 2008; Tyagi et al., 2015). Accordingly, *sufR* enabled persistence in the host by maintaining iron homeostasis, and downregulating genes responsible for iron-sulphur cluster biogenesis (Pandey et al., 2018).

#### 2.5.6 Co-metabolism of multiple nitrogen sources

*M. tuberculosis* appears to not require tight control of its nitrogen sources, as it has evolved the ability to co-metabolize a variety of alternate amino acids as nitrogen sources during infection (Agapova et al., 2019; Borah et al., 2019). It has been established that *M. tuberculosis* utilizes asparagine and glutamate as sole nitrogen sources during persistence (Song et al., 2011; Song and Niederweis, 2012). Originally described as a porin-forming protein, OmpATb (Song et al., 2011), and the asparaginase transporter AnsA (Gouzy et al., 2014) rapidly mediates ammonia secretion from these amino acids, thus neutralizing the acidic phagosomal pH. This may be an adaptive mechanism for pH homeostasis and indicates that asparaginase has evolved two independent key functions, to conduct nitrogen metabolism and counteract the host defense acidification (**Figure 2**, **Supplementary Table 2**).

To circumvent nitric oxide toxicity in macrophages, *M. tuberculosis* acquires nitrate by oxidation of nitric oxide. Upon persister formation, an increase in nitrate reductase occurs, indicating that *M. tuberculosis* utilizes nitrate as an alternative electron acceptor during anaerobic respiration since reduction in electron flow associated with decreasing oxygen levels results in an increased concentration of reduced cofactors, such as NADH (Sohaskey, 2008; Williams et al., 2015). Furthermore, PhoP and DosR regulates expression of nitrite and nitrate reductases for persistence during hypoxia (Singh et al., 2020).

#### 2.5.7 Potassium metabolism tightly regulates the membrane potential

Potassium (K^+^) is a crucial cation required for maintenance of an electrochemical gradient and PMF, and regulation of intracellular pH and osmotic pressure. During phagosome maturation, a change in host ions and K^+^ uptake occurs, thus *M. tuberculosis* tightly controls regulation of its ionic signals *via* the Trk and Kdp K^+^ uptake systems. Disruption of K^+^ homeostasis results in the inability of *M. tuberculosis* to respond to low pH and high chloride levels in BMDMs (MacGilvary et al., 2019), and has been shown to negatively affect *M. tuberculosis* growth rate, as determined by uracil incorporation as a measure of transcriptional activity (Salina et al., 2014).

Interestingly, exposure to low environmental K^+^ repressed iron uptake in *M. tuberculosis*, subsequently exposing bacilli to oxidative stress (MacGilvary et al., 2019). Following K^+^ deficiency, *M. tuberculosis* persisters maintained a stable, yet low abundance of transcripts coding for biosynthetic enzymes or proteins involved in adaptation, repair, and management of transcription initiation. Once reintroduced into K^+^ supplemented media, these mRNA transcripts likely assisted in rapid translation following resuscitation (Ignatov et al., 2015).

#### 2.5.8 Phosphate metabolism and the stringent response

Phosphorous is essential for the synthesis of nucleotides, DNA, RNA and phospholipids, and is acquired from the host *via* the phosphate-specific transporter (Pst) to form phosphate compounds through interaction with the two-component regulatory system SenX3-RegX3 (Rifat et al., 2009; Tischler et al., 2013; Namugenyi et al., 2017). SenX3-RegX3 likely plays a role in virulence during persistence as deletion of *pstA1* constitutively activates RegX3, regardless of phosphate availability, and subsequently triggers the ESX-5 secretion system, resulting in hypersecretion of ESX-5 substrates in Irgm1-deficient mice (Elliott et al., 2019). While the ESX-5 system is required for virulence, tight control in secretion of ESX-5 substrates may limit recognition by the host immune system or prevent secretion of harmful cytokines (Elliott et al., 2019). This may represent a defense mechanism to evade the host adaptive immune response, however it is unknown whether ESX-5 substrates directly assist in phosphate scavenging or whether constitutive secretion of specific ESX-5 antigens may explain the impaired intracellular survival of *M. tuberculosis.*

During nutrient starvation, polyphosphate (polyP) accumulation provides a reservoir of energy and a phosphate donor, and may be the preferential energy store since decreased ATP levels were observed during most stress conditions in guinea pigs (Singh et al., 2013). This was associated with down-regulation of reductive TCA cycle intermediates (succinate, fumarate, malate), and up-regulation of oxidative TCA intermediates, as indicated using ^13^C isotope labelling (Dutta et al., 2019). Additionally, regulation of polyP is dependent on (p)ppGpp levels, as disruption to the stringent response pathway contributed to enhanced antibiotic susceptibility and defective bacterial growth in guinea pig lungs (Singh et al., 2013; Chuang et al., 2015). This suggests that tight regulation of Rel*_Mtb_* and polyP homeostasis is critical for *M. tuberculosis* persister formation following exposure to host-induced stressors and/or antibiotics.

### 2.6 Impact of heterogeneous granuloma lesions on bacterial adaptation

Granulomas develop a spectrum of lesion types, driven by variable cytokine profiles and lesion histopathology (Lin et al., 2013; Martin et al., 2017). Importantly, previous work has shown that granuloma lesions harbor physiologically distinct bacterial populations with varying rates of replication and different metabolic states (Lenaerts et al., 2007; Hoff et al., 2011). Since heterogeneity within lung lesion types influences the bacterial phenotype, this could provide essential information about the microenvironment and how this impacts the bacterial physiology to favor and induce persistence (Dartois, 2014; Irwin et al., 2015; Gregg et al., 2018).

*M. tuberculosis* may exploit the host response by tweaking the inflammatory balance within granulomas. Detailed immunohistochemical analysis of granulomatous lesions from *M. tuberculosis-*infected cynomolgus macaques demonstrated a pro-inflammatory phenotype to be localized to the center of the granuloma (Mattila et al., 2013), which has been shown to contribute to persistence and extracellular survival of *M. tuberculosis* (Tan and Russell, 2015) (**Figure 3**). Contrarily, the tissue within the granuloma surrounding the caseum displays a gradient of anti-inflammatory phenotypes and gradually increasing oxygen tension (Mattila et al., 2013). This suggests that the pathways involved in macrophage metabolism influence the inflammatory signals and effector functions generated in response to infection.

Macrophage heterogeneity attributed to ontogeny and polarization states can further influence macrophage function, and the subsequent progression of varying granuloma types that coexist in individual patients. Using experimental data from nonhuman primates, the development of a computational model was used to investigate the temporal dynamics and spatial organization of M1 and M2 polarized macrophages to determine how cytokine signaling impacts the outcome of infection (Marino et al., 2015). Independent of adaptive immunity, alveolar macrophages in the mouse lung exhibit an M2 phenotype and favor fatty acid oxidation, therefore providing a metabolic and nutritional advantage for intracellular *M. tuberculosis* compared to bacilli residing in interstitial macrophages (Huang et al., 2018). Whilst this mechanism could protect the host against excessive inflammation, it is unclear how *M. tuberculosis* alters the metabolic state of macrophages towards M2 polarization to enhance intracellular persistence and sequestration of host nutrients.

*M. tuberculosis* increases TAG accumulation in the caseum, which contributes to antibiotic tolerance (Daniel et al., 2011; Sarathy et al., 2018), and correlates with the presence of lipid-body positive *M. tuberculosis* in sputum (Sloan et al., 2015). It is thought that foamy macrophages surround the caseum, as their lipid composition resembles the environment encountered in the caseum (Peyron et al., 2008; Kim et al., 2010). Additionally, necrotic death of foamy macrophages would release lipid droplets and cellular debris at the caseum interface (Peyron et al., 2008). Since foamy macrophages have lost their phagocytic ability (Peyron et al., 2008), it is suggested that their formation is exploited by *M. tuberculosis* to enhance cavitation of the granuloma.

### 2.7 Adaptation to antibiotic exposure within the granuloma

Histopathological analysis of lesions indicates that antibiotic treatment induces changes in the granuloma structure, whereby the majority of caseous granulomas evolve to fibrotic or necrotizing tissue that lack proper structure. Hypoxic conditions and poor antibiotic diffusion into the devascularized caseous center lead to heterogeneous susceptibility of bacteria, whereby residual bacterial growth remains in the caseum or acellular rim of necrotic granulomas following treatment in guinea pigs (Lenaerts et al., 2007), rabbits (Sarathy et al., 2018) and macaques (Lin et al., 2014). These bacilli were found to grow as multicellular pellicles, characteristic of biofilms (Ojha et al., 2008; Islam et al., 2012; Wright et al., 2017). It is hypothesized that this cording phenotype contributes to mycobacterial persistence in the host as the abundance of extracellular free mycolic acids during pellicle maturation creates a waxy-layered lipid-rich matrix that harbors and protects drug-tolerant bacilli (Ojha et al., 2008; Islam et al., 2012; Wright et al., 2017).

The host tissue environment and complex granuloma composition likely drives heterogeneous bacterial susceptibility and antibiotic-tolerant persister formation, as some lesions are sterilized prior to antibiotic treatment in macaques (Lin et al., 2014), whilst other lesions may endure following completion of treatment in humans (Malherbe et al., 2016). Differential drug partitioning between cellular and necrotic lesions (Irwin et al., 2016; Sarathy et al., 2016; Zimmerman et al., 2017; Blanc et al., 2018) result in sub-inhibitory antibiotic concentrations, thereby impacting the rate of sterilization and treatment success. The prodrugs isoniazid and pyrazinamide additionally require bioactivation by *M. tuberculosis* KatG and PncA, respectively, to be functional. Whilst decreased expression of KatG is associated with isoniazid-treated persisters (Wakamoto et al., 2013; Kurthkoti et al., 2017), genes other than *pncA*, such as *sigE* and *panD* are implicated in pyrazinamide resistance (Thiede et al., 2022). It remains to be determined whether the latter genes mediate tolerance in *M. tuberculosis*. Inhibition of F_1_F_0_-ATP synthase following treatment with bedaquiline involves rapid metabolic adaptation by *M. tuberculosis*, whereby multiple metabolic pathways associated with the *dosR* dormancy regulon, lipid homeostasis and cell wall remodeling contribute to antibiotic tolerance (Peterson et al., 2016).

Persister formation and associated antibiotic tolerance may be triggered by host exposure, independent of antibiotic exposure. Increased antibiotic tolerance may thus be attributed to a combination of alternative metabolic pathways being utilized that may not be bioactivated, and inconsistent drug distribution attributed to lesional heterogeneity. In previous work from our group, macrophage uptake resulted in enrichment for non- or slowly replicating *M. tuberculosis*. Treatment with D-cycloserine revealed that this population is highly enriched for persisters, based on its antibiotic tolerant phenotype (Mouton et al., 2016). This is in line with work from Jain et al. (2016) who observed the presence of persisters in sputum from TB patients prior to antibiotic treatment. Vilchèze et al. (2017) additionally demonstrated that compounds that enhanced respiration and induced ROS in *M. tuberculosis* persisters prevented *M. tuberculosis* from entering a low metabolic state and rendered mycobacteria susceptible to bactericidal antibiotics (Vilchèze et al., 2017). This suggests that host pressures drive formation of antibiotic-tolerant persisters, and may be exploited by targeting processes required during maintenance of persistence in the host.

To mimic intracellular confinement and spatially monitor the impact of phagosomes on *M. tuberculosis* antibiotic-tolerant persister formation, space-confined cell culture chambers (Luthuli et al., 2015) and zebrafish larval models (Adams et al., 2011) have been exploited. This revealed differential progression of lesions in response to antibiotic treatment. Following macrophage uptake, drug tolerance arose within a few days, whereby antibiotic-tolerant persisters residing in macrophages exploited granulomas for their expansion or migrated to disseminate infection (Adams et al., 2011; Luthuli et al., 2015). Recent approaches to tracking the fate of individual lesions provides insight into the heterogeneous granuloma formation and size, and lesion-specific dissemination (Martin et al., 2017; Gregg et al., 2018; Cicchese et al., 2020), which may guide our understanding of how specific tissue environments influence responses to antibiotics.

## 3 Phenotypic adaptation of *M. tuberculosis* to host-associated stressors

The varying microenvironments that *M. tuberculosis* colonizes greatly enhance phenotypic adaptations that enable host evasion and promotes intracellular survival and persistence. A major adaptation is an altered growth rate. A slower growth rate has been associated with modifications to the mycobacterial cell wall architecture, whereby *M. tuberculosis* reconstructs its surface lipid and mycolic acid structure by undergoing mycolic acid biosynthesis and cell wall thickening (Hampshire et al., 2004; Rohde et al., 2012; Vilchèze and Kremer, 2017; Zimmermann et al., 2017; Baker and Abramovitch, 2018). An altered colony morphology (Salina et al., 2014) and cell length distribution observed in sputum (Vijay et al., 2017b) have been further shown to influence heterogeneity in metabolism, antibiotic sensitivity and response to stressors.

### 3.1 Structural adaptation

Prolonged periods under multiple stress conditions drastically increases the number of intracellular lipid bodies *in vitro* (Deb et al., 2009) and in sputum (Garton et al., 2008; Vijay et al., 2018), whereby a correlation between TAG accumulation and loss of acid fastness is observed (Deb et al., 2009; Daniel et al., 2011). Interestingly, transcriptomic analyses of these bacilli revealed downregulation of *kasB*, one of two *M. tuberculosis* genes encoding distinct ß-ketoacyl-ACP synthases, during persistence (Deb et al., 2009; Vilchèze et al., 2014). Deletion of *kasB* led to alterations in mycolic acid structure by producing mycolic acids that were two to four carbons shorter, and resulted in the loss of cording and acid-fastness (Gao et al., 2003; Bhatt et al., 2007). This may negatively affect cell wall permeability since these mycolic acids were not synthesized in dense bundles (Yamada et al., 2012). The precise mechanism resulting in loss of acid fastness is however not well understood, and it is uncertain whether retention of the acid-fast stain is hindered or whether reorganization of the cell wall components prevents access of the stain.

The current acid-fast staining approach is dependent on mycolic acid chain length and structure, which has been shown to substantially differ in the cell wall of *M. tuberculosis* (Barry et al., 1998; Beken et al., 2011). Persisters residing in the caseum frequently go undetected and appear acid-fast negative in guinea pigs (Hoff et al., 2011), highlighting the inconsistencies of acid-fast staining. Histological representation of various lung lesions from patients non-compliant to treatment display heterogeneous morphology and distribution of acid-fast bacilli. Acid-fast bacilli were largely located at the granuloma surface, with connection to airways, whereas acid-fast bacilli in necrotizing granulomas were scarcely detected (Kaplan et al., 2003; Lenaerts et al., 2007). Interestingly, following resuscitation of *M. tuberculosis* persisters generated *in vitro*, upregulation of genes responsible for fatty acid and mycolic acid biosynthesis was observed (Du et al., 2016; Salina et al., 2019), whilst pathways involved in lipid uptake and catabolism were down-regulated (Du et al., 2016). This characteristic may reflect differences in the cell wall components and lipid profiles prior to resuscitation; it is thus intriguing whether these bacilli regained their acid-fastness.

Nutrient availability further influences variations in morphology, whereby phosphate- (Rifat et al., 2014) or potassium-starved bacilli (Salina et al., 2014) display a more elongated, or spherical and shorter morphology, respectively. This may alter the ultrastructure and could explain the loss in acid-fastness of *M. tuberculosis* persisters. The enhancement in surface-to-volume ratio for elongated cells may be advantageous in sequestering additional nutrients from its environment.

### 3.2 Cell division and growth rate

Asymmetric cellular division is commonly observed in mycobacteria during cell elongation and division (Aldridge et al., 2012). As bacterial cells divide, the progeny inherits an ‘old pole’ generated from previous cell divisions, and a ‘new pole’ from the most recent cell division. Cell length heterogeneity introduced by asymmetrical cell division has been associated with differential susceptibility to host- and antibiotic-induced stressors (Aldridge et al., 2012; Vijay et al., 2017a). Asymmetry-promoting proteins LamA and Wag31 regulate polar growth and cell elongation at each pole (Rego et al., 2017). Cells inheriting the old pole generate longer cells with faster elongation rates than cells that inherit the new pole (Aldridge et al., 2012; Richardson et al., 2016; Nair et al., 2019; Vijay et al., 2017a). Unequal partitioning of cells, with a stronger bias towards daughter cells inheriting the old pole, display higher tolerance to host and antibiotic stress (Richardson et al., 2016; Nair et al., 2019). Similarly, multidrug-resistant (MDR) strains were observed to increase their cell length distribution in macrophages (Vijay et al., 2017b), whilst shorter cells with slower elongation rates were more antibiotic susceptible (Aldridge et al., 2012; Nair et al., 2019). Computational tools that cytologically profile *M. tuberculosis* response to antibiotic treatment (Smith et al., 2020) may further be adapted to predict whether specific cell morphologies prior to treatment influence treatment outcome.

Synthesis of cell envelope components such as peptidoglycan and arabinogalactan varies in polar localization (Botella et al., 2017), thus asymmetrical division may impact on cell envelope assembly, resulting in distinct localization of cellular or metabolic machinery near poles that will allow for efficient growth. Repeated inheritance of the old poles at cellular division has been linked to limited growth rates, increased antibiotic susceptibility and cell death (Dhar and McKinney, 2007; Aldridge et al., 2012). This may be attributed to partitioning of damaged cell components, which would describe replicative aging and cell death. However, whether pole age influences survival or entry into persistence is unknown.

Alternative carbon sources (Priestman et al., 2017) or nutrient starvation (Hayashi et al., 2018) may further reflect variations in growth rate and cell length. Oxidative stress and iron deficiency have been shown to increase cell length heterogeneity in sensitive and MDR strains (Vijay et al., 2017b). A higher level of NADH oxidase in shorter cells generates significantly higher hydroxyl radical levels *via* the Fenton reaction. Since this occurs to a lesser extent in normal/long-length cells, this inherent predisposition could offer a survival advantage attributed to differential antibiotic susceptibility, and may provide a mechanism underlying tolerance (Vijay et al., 2017b). Interestingly, shorter cells possessed a lower buoyant density fraction in Percoll gradient, indicative of a high lipid content. The presence of lipid-rich membrane vesicles on the bacilli cell surface of shorter cells may indicate molecular differences to normal/long-length cells (Vijay et al., 2017a). Regardless of the growth media, the short and normal/long-length cells comprised ~ 10% and ~ 90% of the population, respectively, *in vitro* and in clinical isolates (Vijay et al., 2017a). Stressed *M. tuberculosis* additionally distributes irreversibly oxidized proteins asymmetrically (Vaubourgeix et al., 2015). This may represent an inherent mechanism for regulation of mycobacterial cell growth, further enhancing population heterogeneity, and requires further investigation.

## 4 Conclusion

Bacterial phenotypic heterogeneity signifies a survival strategy to allow rapid adaptation and persister formation in response to altering conditions (Ryan et al., 2010). *M. tuberculosis* is simultaneously exposed to multiple host-related stressors, contributing to the spectrum of adaptive responses that induce phenotypically heterogeneous subpopulations. Research highlights the induction of overlapping adaptive responses that may assist *M. tuberculosis* persisters to flourish in various microenvironments within the host, and further contributes to antibiotic tolerance (Kurthkoti et al., 2017; Huang et al., 2018; Pisu et al., 2020). *M. tuberculosis* appears to exploit macrophages and granuloma formation for its expansion. Therefore, utilizing animal models that reflect the diversity within lesion phenotypes as observed in humans can divulge how host interactions drive bacterial phenotypic heterogeneity, and subsequent disease progression (Driver et al., 2012; Martin et al., 2017). Many technical challenges are however associated with investigation of *M. tuberculosis* persisters, thus approaches involving flow cytometry and high-content imaging-based techniques in combination with *M. tuberculosis*-specific probes could improve the characterization of persisters (Parbhoo et al., 2020). This could highlight specific mechanisms utilized by *M. tuberculosis* for adaptation to the host environment; understanding and exploiting such pathways may highlight key mechanisms utilized by *M. tuberculosis* to maintain persistence, possibly providing a target for therapeutic intervention.

## Author contributions

TP researched data, constructed figures, wrote and edited the manuscript. JM and SS critically reviewed the manuscript and provided insightful discussions and ideas. All authors contributed to the article and approved the submitted version.

## Funding

This work was supported by funding from the South African Medical Research Council (SA MRC), and the South African National Research Foundation (NRF). SS is funded by the South African Research Chairs Initiative of the Department of Science and Technology and NRF of South Africa, award number UID 86539. TP was funded by the Deutscher Akademischer Austauschdienst and NRF of South Africa, award number UID 111868. This work was supported by the GCRF Networks in Vaccines Research and Development VALIDATE Network which was co-funded by the MRC and BBSRC (ref MR/R005850/1). This UK funded award is part of the EDCTP2 programme supported by the European Union.

## Conflict of interest

The authors declare that the research was conducted in the absence of any commercial or financial relationships that could be construed as a potential conflict of interest.

## Publisher’s note

All claims expressed in this article are solely those of the authors and do not necessarily represent those of their affiliated organizations, or those of the publisher, the editors and the reviewers. Any product that may be evaluated in this article, or claim that may be made by its manufacturer, is not guaranteed or endorsed by the publisher.
