# Supplementary material for: Phenotypic adaptation of Mycobacterium tuberculosis to host-associated stressors that induce persister formation
Source: Front Cell Infect Microbiol. 2022 Sep 27;12:956607. doi: 10.3389/fcimb.2022.956607 (PMC9551238; doi:10.3389/fcimb.2022.956607)
Supplement: Supplementary file 1 [file Table_1.docx]

| Supplementary Table 1: Pathways involved in adaptation to stress and entry into persistence. | | | | | | |
| --- | --- | --- | --- | --- | --- | --- |
|  | **Gene name** | **Locus** | **Protein** | **Phenotype associated with adaptation** | **Functional effect of gene mutation** | **References** |
| Phagosome maturation & acidification | *phoP* | Rv0757 | Two-component regulatory system positive transcriptional regulator | - Mediates adaptation to early hypoxic response in combination with DosR and nitrogen metabolism - Maintains redox homeostasis by controlling expression of *ald* - PhoP in combination with isocitrate lyase regulates synthesis of the cell-envelope lipids, sulfolipid and acylated trehaloses - Regulates induction of ESX-1 lipid biosynthesis, thereby promoting ESAT-6 export - Putative role in the cording phenotype | Attenuated for virulence in macrophages and mice; attenuated during hypoxia irrespective of nitrogen availability; impaired synthesis of methyl-branched fatty-acids; alteration to major cell wall components, no ESAT-6 export | Gonzalo-Asensio *et al.,* 2008; Singh *et al.,* 2020; Walters *et al.,* 2006 |
|  | *phoPR* | Rv0757, Rv0758 | Two-component regulatory system | - Sensor histidine kinase (PhoR) senses and responds to external pH by regulating expression of *aprABC* - Regulates heat shock-responsive genes (*acr2*, *groEL2*) and *whiB3*-regulated redox-sensing in response to low pH | Attenuated for growth and virulence in macrophages and mice; altered cell wall | Feng *et al.,* 2018; Sevalkar *et al.,* 2019; Solans *et al.,* 2014; Walters *et al.,* 2006 |
|  | *esat-6 (esxA)* | Rv3875 | 6-kDa Early Secretory Antigenic Target | - Virulence factor for pathogenesis; ESX-1 and PhoPR dependent - Perforation of the phagosome membrane during acidic pH by increasing membrane fluidity; prevents phagolysosomal fusion; assists in cytosolic translocation | Attenuated for virulence; decreased membrane lytic activity and cytosolic translocation in macrophages | Augenstreich *et al.,* 2017; Quigley *et al.,* 2017; Ray *et al.,* 2019 |
|  | *aprABC* | MT2466, MT2467,  Rv2396 | Acid and phagosome regulated | - PhoPR-dependent induction during acidic conditions - Putative role in TAG and PDIM accumulation; regulates carbon flux into cell wall lipids | Defective lipid metabolism | Abramovitch *et al.,* 2011 |
|  | *virS* | Rv3082c | Virulence-regulating transcriptional regulator | - Regulates cytoplasmic pH during acidic pH; phagosome maturation arrest - Regulates genes involved in fatty acid oxidation and biosynthesis, thereby contributing to cell wall remodelling | Altered colony morphology and cell wall structure attributed to altered lipid content; decreased survival in macrophages | Singh *et al.,* 2005, 2019 |
|  | *ompATb* | Rv0899 | Outer membrane protein A | - Generates rapid ammonia secretion to neutralize the acidic pH; strongly induced during growth in acidic pH and macrophages | Unable to maintain pH homeostasis, impaired growth during acidic pH | Raynaud *et al.,* 2002; Song *et al.,* 2011 |
|  | *pckA* | Rv0211 | Phosphoenol-pyruvate carboxykinase | - Regulates metabolism at acidic pH by either replenishing methyl citrate cycle intermediates in the TCA cycle or gluconeogenic pathway - Metabolism of fatty acids as a carbon source | Attenuated virulence and growth in macrophages and mice; unable to grow in media containing fatty acids; over accumulation of methyl citrate cycle and TCA intermediates; increased NADH/NAD^+^ ratio | Marrero *et al.,* 2010; Quinonez *et al.,* 2022 |
|  | **Gene name** | **Locus** | **Protein** | **Phenotype associated with adaptation** | **Functional effect of gene mutation** | **References** |
| Oxidative stress | *whiB3* | Rv3416 | Redox- and pH-responsive transcriptional regulator | - Cytoplasmic iron-sulphur cluster redox and pH-responsive transcriptional regulator involved in phagosome maturation arrest - Induces DNA repair genes - Regulates production of the low molecular weight thiols, ergothionine and mycothiol during nutrient starvation and acid stress, respectively - Facilitates metabolic switching to fatty acids by catabolizing TAG for synthesis of the cell-envelope lipids, sulfolipid, acylated trehaloses, PDIM | Attenuated in macrophages and guinea pigs; redox stress; altered cell wall lipid composition and morphology; impaired ESX-1 secretion; defective bioenergetic metabolism | Buchmeier *et al.,* 2006; Mehta *et al.,* 2016; Richard-Greenblatt *et al.,* 2015; Saini *et al.,* 2016 |
|  | *clpB* | Rv0384c | Chaperone protein ClpB | - Facilitates disaggregation and refolding of damaged or irreversibly oxidized proteins (IOPs) - Asymmetrically distributes IOPs between progeny introduces variation in growth rate and antibiotic susceptibility | Survival defect and aberrant cellular morphology during hypoxia, heat and oxidative stress; accumulation of IOPs in macrophages and mice | Tripathi *et al.,* 2020; Vaubourgeix *et al.,* 2015 |
|  | *katG* | Rv1908c | Catalase peroxidase | - Maintains redox and pH homeostasis using antioxidant mechanisms by catalysing conversion of damaging hydrogen peroxide to water and oxygen - Heme enzyme, downregulated upon iron starvation | Attenuated virulence in macrophages and mice; sensitivity to redox stress; high-level resistance to INH | Kurthkoti *et al.,* 2017a; Vilchèze *et al.,* 2020 |
|  | *mazG* | Rv1021 | Nucleoside triphosphate pyrophospho-hydrolase | - Pyrimidine-specific housekeeping enzyme that degrades and prevents incorporation of oxidized deoxynucleotides into DNA, prevents DNA mutagenesis - Transcriptionally activates RelA in response to oxidative stress | Incorporates oxidized pyrimidine nucleotides into DNA via error-prone DNA polymerase DnaE2; increased CG to TA mutations; hypersusceptibility to RNS, induces lethal dsDNA breaks | Fan *et al.,* 2018; Lyu *et al.,* 2013; Shi *et al.,* 2019 |
|  | *sodA* | Rv3846 | Superoxide dismutase A | - Converts damaging superoxide radicals to hydrogen peroxide - Iron-containing enzyme; increased secretion during nutrient starvation | Severely growth attenuated in mice | Edwards *et al.,* 2001; Nambi *et al.,* 2015; Piddington *et al.,* 2001 |
| Hypoxia | *dosR* | Rv3133c | Dormancy survival regulator | - Regulates response to nitric oxide, hypoxia, redox stress and DNA damage - Delays the onset of the host adaptive immune response attributed to altered antigen presentation - Regulates biosynthesis of TAG and is involved in reactivation of growth | Attenuated in macaques and C3HeB/FeJ mice; unable to regulate NADH/NAD^+^ ratio | Gautam *et al.,* 2015; Mehra *et al.,* 2015 |
|  | *sigB, sigE, SigH* | Rv2710, Rv1221, Rv3223c | RNA polymerase sigma factor | - Sigma factor; mediates adaptation to early hypoxic response independent of carbon source - Regulates genes involved in stress adaptation and cell wall stress | Prolonged lag phase when exposed to oxygen, decreased bacterial survival | Du *et al.,* 2016; Dutta *et al.,* 2010; Fontán *et al.,* 200) |
|  | **Gene name** | **Locus** | **Protein** | **Phenotype associated with adaptation** | **Functional effect of gene mutation** | **References** |
|  | *clgR* | Rv2745c | Transcriptional regulatory protein | - Adaptation to hypoxia in the presence of fatty acids; regulates genes involved in stress adaptation - Maintenance of membrane integrity and tolerance to surface stress - Assists in reactivation of growth | Sensitive to redox stress; decreased bacterial survival in macrophages | Datta *et al.,* 2015; McGillivray *et al.,* 2014 |
| Growth rate | *lamA* | Rv2198c | Loss of asymmetry mutant A | - Member of the mycobacterial division complex; promotes asymmetrical growth by inhibiting cell wall synthesis at the new pole, thus prolonging its elongation | Decreases asymmetrical polar growth by adding new cell wall material at similar rates between the old and new pole | Rego *et al.,* 2017 |
|  | *wag31* | Rv2145c | Cell wall synthesis protein | - Maintains cell morphology and cell wall structure by localizing peptidoglycan synthesis to the cell poles - Protection from oxidative stress | Morphological defect, cell becomes spherical shaped and susceptible to lysis | Jani *et al.,* 2010; Kang *et al.,* 2008; Mukherjee *et al.,* 2009 |
|  | *kasB* | Rv2246 | β-ketoacyl- acyl-carrier-protein synthase B | - Serine/threonine kinase-dependent phosphorylation and regulation of mycolic acid chain length and acid-fast staining | Attenuated growth; production of shorter mycolic acids, loss of acid-fast staining and cording, alters cell morphology and cell wall permeability | Bhatt *et al.,* 2007; Vilchèze *et al.,* 2014 |

| Supplementary Table 2: *M. tuberculosis* adaptation in response to nutrient deprivation | | | | | |
| --- | --- | --- | --- | --- | --- |
|  | **Element** | **Protein/Enzyme** | **Phenotype associated with adaptation** | **Functional effect of gene mutation** | **References** |
| Amino acids  Amino acids | *Rel*_Mtb_ | Stringent response | - Regulates and catalyses the synthesis of (p)ppGpp (hyperphosphorylated guanine nucleotide-based intracellular signalling molecules) during amino acid deprivation, hypoxia and oxidative stress - Conserves energy by inhibiting RNA synthesis during amino acid deprivation | Metabolic profile resembled exponentially growing bacilli in nutrient-rich media, unable to slow replication rate, possessed reduced antibiotic tolerance, incapable of inducing the stringent response and polyP accumulation | Dutta *et al.,* 2019 |
|  | *lrpA* | leucine-responsive regulatory protein | - Amino acid homeostasis; regulates amino acid catabolism and anabolism; metabolism of L-lysine and glutamate - Regulates synthesis of (p)ppGpp and *pknG* | Decreased intracellular amino acid metabolism | Duan *et al.,* 2016; Rieck *et al.,* 2017a |
|  | *pknG* | Protein kinase G | - Serine/threonine protein kinase; regulates amino acid metabolism, redox homeostasis, and cell envelope biosynthesis - SecA2-dependent export; prevents phagolysosomal fusion by inhibiting Rab5 dissociation and Rab7 recruitment | Attenuated growth in guinea pigs; dysregulation of intracellular amino acid levels and utilization of host-derived carbon sources; oxidative stress and increased mycothiol levels; defects in inhibiting phagosome maturation | Khan *et al.,* 2017; Rieck *et al.,* 2017; Zulauf *et al.,* 2018 |
|  | *lat* | Lysine ε-aminotransferase | - Vitamin B_6_-dependent lysine catabolic enzyme - Degrades lysine accumulated from methionine and threonine deprivation - Replenishes the amino acid pool by catalysing transamination and deamination reactions | Decreased transcription/abundance of intracellular amino acids, possibly attributed to the resulting down-regulation of *lrp* and (p)ppGpp | Duan *et al.,* 2016; Hasenoehrl *et al.,* 2019 |
|  | *metX* (*metA*) | Homoserine transacetylase | - Scavenges biosynthetic intermediates from the host for methionine, threonine, and S-adenosylmethionine (SAM) biosynthesis | Attenuated; rapid metabolic shutdown and clearance from mice attributed to inhibition of SAM-dependent methyltransferase reactions | Berney *et al.,* 2015; Hasenoehrl *et al.,* 2019 |
|  | *ald* | L-alanine dehydrogenase | - Strongly induced by NO; maintains redox balance possibly by preventing growth until the NADH/NAD^+^ redox balance has shifted towards an oxidized form, by interaction with the glyoxylate and methylcitrate cycle - Decreases the abundance of toxic glycolytic intermediates during hypoxia by reductive amination of pyruvate to alanine, or glyoxylate to glycine via NADH oxidation - Maintains cell wall homeostasis by conversion of L-alanine to D-alanine - Putative role in the resuscitation of persisters | Inability to oxidize NADH to NAD^+^ resulted in enhanced NADH levels; delayed recovery from hypoxia.  Ald inhibitors displayed disruption to the redox balance and the cytochrome bcc/aa3 complex | Awasthy *et al.,* 2012; Dey *et al.,* 2016; Eoh and Rhee, 2013; Jeong *et al.,* 2018; Reshma *et al.,* 2016; Saxena *et al.,* 2015 |
|  | **Element** | **Protein/Enzyme** | **Phenotype associated with adaptation** | **Functional effect of gene mutation** | **References** |
| Vitamins | *metH* and *metE* | Vitamin B_12_-dependent and B_12_-independent methionine synthase | - β-oxidation of fatty acids; detoxification of propionyl-CoA via the methylmalonyl-CoA and methyl citrate cycle is a vitamin B12-dependent and B12-independent mechanism, respectively - MetH behaves as a B_12_-responsive riboswitch, suppressing transcription of *metE* during B_12_ availability | Attenuated growth attributed to suppression of methionine synthase activity  $\Delta$*metH*: Riboswitch-mediated suppression of *metE* when exposed to B_12_, increased mutations in the B_12_ riboswitch | Savvi *et al.,* 2008; Warner *et al.,* 2007 |
|  | *bacA* | ABC-transporter | - Proposed ABC transporter for scavenging host vitamin B_12_ - Cholesterol metabolism; assimilation of propionyl-CoA dependent on Icl | Inhibited vitamin B_12_ transport | Gopinath *et al.,* 2013 |
|  | *dfrA* | Dihydrofolate reductase | - Coupled with NADPH, converts folate to its reduced forms, tetrahydrofolate (THF) and 5-methyltetrahydrofolate (5-MTHF), required for production of nucleic acids, purines, thymidine, methionine, glycine, serine, homocysteine and SAM | Inhibitors of dfrA decreased methionine and reduced folate intermediates; disrupted SAM-dependent methyltransferase activity; decreased production of mycolic acids | Chakraborty *et al.,* 2013; Nixon *et al.,* 2014; Zheng *et al.,* 2013 |
|  | *bioA* | 7,8-diaminopelargonic acid aminotransferase | - Essential cofactor involved in the biosynthesis of Biotin (Vitamin B_7_) - Involved in fatty acid biosynthesis, amino acid metabolism, replenishment of TCA intermediates | Attenuated growth and virulence in guinea pigs; rapid decline in posttranslation biotinylation of pyruvate carboxylase and acyl-CoA carboxylase; compromised cell envelope; loss in acid-fastness | Kar *et al.,* 2017; Lazar *et al.,* 2017; Park *et al.,* 2011 |
|  | *panC* and *panD* | Pantothenate biosynthesis | - Biosynthesis of Vitamin B_5_, required for synthesis of coenzyme A (CoA) and acyl carrier proteins needed for biosynthesis of polyketide and cholesterol, fatty-acid metabolism, and the TCA cycle | Attenuated in mice; auxotrophic for pantothenate; impairment in lipid biosynthesis | Sambandamurthy *et al.,* 2002 |
| Phosphate | *pstA1* | Phosphate-specific transporter | - High-affinity, low velocity membrane-spanning transporters; PstA1 regulates gene expression during phosphate limitation, whilst PstA2 enhances tolerance to acidic pH - Regulates the two-component regulatory system SenX3-RegX3, to sense and regulate response to phosphate limitation, respectively | Attenuated growth and virulence in mice and activated macrophages; *pstA1* deletion constitutively activates RegX3 and triggers hypersecretion of ESX-5 substrates regardless of phosphate availability | Namugenyi *et al.,* 2017; Rifat *et al.,* 2009; Tischler *et al.,* 2016, 2013 |
|  | *ppk-1* | Polyphosphate kinase 1 | - Synthesizes polyphosphate (polyP) [polymer containing hundreds of phosphate residues linked via high-energy phosphoanhydride bonds] through ATP hydrolysis; provides a reservoir of energy and a phosphate donor - PolyP accumulation controls expression of *Rel*_Mtb_, drives synthesis of (p)ppGpp - Modulates protein synthesis, lipid metabolism, cell wall remodelling | Attenuates growth in macrophages, impaired virulence in guinea pigs; enhanced antibiotic susceptibility | Chuang *et al.,* 2015; Dutta *et al.,* 2019; Elliott *et al.,* 2019; Sanyal *et al.,* 2013; Singh *et al.,* 2013; Thayil *et al.,* 2011 |
|  | *ppk-2* | Polyphosphate kinase 2 | - Catalyses polyP-dependent phosphorylation of ADP to ATP - Controls intracellular polyP levels and nucleotide pool | Attenuates growth in mice; excessive accumulation of polyP | Chuang *et al.,* 2013; Sureka *et al.,* 2009 |
|  | **Element** | **Protein/Enzyme** | **Phenotype associated with adaptation** | **Functional effect of gene mutation** | **References** |
| Iron | *ideR* | Iron-dependent regulator | - Transcriptionally regulates iron homeostasis; positively regulates iron storage genes (*bfrA, bfrB)* during iron limitation, acts as a negative regulator upon sufficient iron - Regulates biosynthesis of Fe^3+^-specific siderophores (carboxymycobactin & mycobactin) for iron chelation from insoluble and protein-bound iron | Attenuated growth in macrophages and mice; defective siderophore synthesis and uptake or inability to store excess iron resulted in hypersensitivity to oxidative stress | Pandey and Rodriguez, 2014, 2012; Ryndak *et al.,* 2010; Wells *et al.,* 2013 |
|  | *mbtE* | Mycobactin synthetase | - Synthesis of the high-affinity iron-binding siderophore, mycobactin, required for iron uptake | Attenuated growth and virulence in guinea pigs and macrophages irrespective of iron availability; altered colony morphology and cell wall permeability; unable to synthesize mycobactin | Reddy *et al.,* 2013; Tufariello *et al.,* 2016 |
|  | *esx-3* | Type VII secretion system | - Secretion system required for iron homeostasis and mycobactin-mediated iron acquisition; IdeR-dependent - Export of esx-3-encoded substrates is iron-dependent | Attenuated growth in macrophages; defective in acquisition of bound iron from siderophores; overproduction of mycobactin | Kurthkoti *et al.,* 2017; Serafini *et al.,* 2013; Siegrist *et al.,* 2009; Tufariello *et al.,* 2016 |
|  | *hupB* | Histone-like protein | - Positively regulates siderophore biosynthesis in response to IdeR - Putative role in siderophore export - DNA-binding protein; stabilizes DNA architecture to prevent denaturation | Attenuated growth in macrophages; decreased biosynthesis and abundance of siderophores | Pandey *et al.,* 2014 |
| Sulphur | *cysH* | 5'-adenosinephosphosulphate reductase | - Maintenance of redox reactions; produces reduced-sulphur-containing metabolites and iron-sulphur cluster biogenesis - Protection from oxidative and nitrosative stress - SAM and mycothiol biosynthesis; reverse transsulfuration | Auxotrophic for cysteine and methionine; attenuated growth and virulence in mice; unable to synthesize mycothiol; induced a long-lasting and irreversible oxidative shift | Senaratne *et al.,* 2006 |
|  | *sufR* | *suf* operon repressor | - Upregulated during low iron and oxidative stress; mediates intracellular iron homeostasis by downregulating genes responsible for iron-sulphur cluster biogenesis | Increased expression of the suf operon led to sequestration of excess iron and oxidative damage | Pandey *et al.,* 2018 |
|  | *iscS* | Cysteine desulfurase | - Iron-sulphur cluster biogenesis; repair of oxidatively damaged iron-sulphur clusters | Hypersensitive to oxidative stress; impaired iron-sulphur cluster assembly | Rybniker *et al.,* 2014 |
|  | **Element** | **Protein/Enzyme** | **Phenotype associated with adaptation** | **Functional effect of gene mutation** | **References** |
| Carbon & lipid metabolism | *icl1* | Isocitrate lyase/ methylisocitrate lyase | - Metabolism of fatty acids through the glyoxylate shunt; accumulates cytoplasmic lipid bodies, particularly TAG, for use as a carbon and energy source during nutrient limitation - Maintains the PMF and ATP production; regulates metabolism at acidic pH - Metabolism of propionyl-CoA via the methylmalonyl pathway in the presence of vitamin B_12_ | Attenuated virulence in mice and activated macrophages; accumulated cholesterol-derived propionyl-CoA | Eoh and Rhee, 2013; McKinney *et al.,* 2000; Prosser *et al.,* 2017 |
|  | *mmpL11* | Mycobacterial membrane protein large 11 | - Cell wall biogenesis; transports TAG and mycolate wax esters to the cell surface for incorporation into the cell envelope - Mediator of biofilm formation | Attenuated growth; decreased levels of mycolic acid and long-chain TAGs transported to the cell envelope; altered cell morphology, size and lipid composition | Pacheco *et al.,* 2013; Wright *et al.,* 2017 |
|  | *tgs1* | Triacylglycerol synthase 1 | - Induces accumulation of host lipids and TAG-derived fatty acids for intracellular storage when exposed to multiple stressors | Inability to utilize and accumulate fatty acids from TAG for lipid metabolism; antibiotic susceptible | Baek *et al.,* 2011; Daniel *et al.,* 2011; Deb *et al.,* 2009 |
|  | *pykA* | Pyruvate kinase | - Involved in carbon co-catabolism of glycolytic and gluconeogenic substrates - Metabolism of short-chain fatty acids | Attenuated growth in the presence of multiple carbon sources and short-chain fatty acids; accumulation of toxic glycolytic substrates | Noy *et al.,* 2016 |
| Potassium | *ceoBC* | Trk K^+^ uptake system | - Low-moderate affinity K^+^ uptake system - Senses and responds to environmental ionic changes, maintains ionic homeostasis of K^+^, Cl^-^ and intracellular pH | Attenuated growth in macrophages and mice; decreased K^+^ uptake, unable to maintain ionic homeostasis | MacGilvary *et al.,* 2019 |
|  | *kdpA* | Kdp K^+^ uptake system | - ATP-dependent, high-affinity potassium pump - Induced during low K^+^, maintains intracellular pH and ATP homeostasis | Hyperpolarization of the membrane potential, increased PMF and ATP levels; diffusion of large hydrophobic molecules may be disrupted | Liu *et al.,* 2020 |
|  | **Element** | **Protein/Enzyme** | **Phenotype associated with adaptation** | **Functional effect of gene mutation** | **References** |
| Nitrogen | *ansA* | Asparaginase transporter | - Assimilates nitrogen and neutralizes pH through asparagine hydrolysis and ammonia release, respectively, from glutamate and glutamine - Involved in phagosome acidification arrest | Attenuated growth and virulence in macrophages and mice; highly susceptible to acidic pH | Gouzy *et al.,* 2014 |
|  | *argB*, *argF* | Acetyl glutamate kinase, ornithine carbamoyl transferase | - Involved in the arginine biosynthesis pathway - Mediates adaptation to early oxidative damage response, - Induction of genes involved in cell envelope stress and remodelling, DNA repair, and mycothiol, ergothionine, and iron-sulphur cluster biosynthesis | Attenuated in mice; auxotrophic for arginine; extensive ROS accumulation and oxidative damage; DNA damage | Tiwari *et al.,* 2018 |
|  | *narGHIJ* | Nitrate reductase | - Adaptation to nitrate respiration enables protection from acid stress, hypoxia, nitrosative stress, and iron depravation during anaerobic respiration - Requires molybdenum cofactor (MoCo) during persistence; utilized by numerous enzymes to catalyse redox reactions (NADH/NAD^+^) recycling and ATP production | $\Delta$*narG*,$\Delta$*narGH*: cells unable to undergo nitrate respiration irrespective of exogenous nitrate addition; ATP synthesis diminished; susceptible to acidic pH | Cunningham-Bussel *et al.,* 2013; Tan *et al.,* 2010; Williams *et al.,* 2015 |
|  | *narK2* | Nitrate/nitrite transporter | - Regulated by DosR regulon; strongly upregulated during hypoxia and nitric oxide exposure to rapidly induce NarGHIJ - Import and transport of nitrate, and export of nitrite across the cell membrane for induction of NarGHIJ | Low level nitrate reductase activity observed attributed to diffusion; lack of nitrate transport | Giffin *et al.,* 2012; Sohaskey, 2008 |
|  | *mobA* | Molybdenum cofactor guanylyltransferase | - Involved in the conversion of MoCo to *bis*-molybdopterin guanine dinucleotide (*bis*-MGD) to support nitrate assimilatory and reductase activity - Enables NarGHI-dependent growth on nitrate as a sole nitrogen source | Loss of assimilatory and respiratory nitrate reductase activity; impairs persistence and survival in guinea pigs | Williams *et al.,* 2015 |
